# Management of massive diffuse alveolar hemorrhage in a child with systemic lupus erythematosus

**DOI:** 10.1186/s40560-015-0076-5

**Published:** 2015-03-07

**Authors:** Dai Kimura, Samir Shah, Mario Briceno-Medina, Shyam Sathanandam, Brent Haberman, Jie Zhang, Linda Myers, TK Susheel Kumar, Christopher Knott-Craig

**Affiliations:** Division of Pediatric Critical Care Medicine, Department of Pediatrics, Le Bonheur Children’s Hospital/University of Tennessee Health Science Center, 50 N. Dunlap St, TN Memphis, 38103 USA; Department of ECMO/Apheresis, Le Bonheur Children’s Hospital/University of Tennessee Health Science Center, 50 N. Dunlap St, Memphis, TN USA; Department of Cardiology, Le Bonheur Children’s Hospital/University of Tennessee Health Science Center, 50 N. Dunlap St, Memphis, TN USA; Department of Pulmonology, Le Bonheur Children’s Hospital/University of Tennessee Health Science Center, 50 N. Dunlap St, Memphis, TN USA; Department of Pathology, Le Bonheur Children’s Hospital/University of Tennessee Health Science Center, 50 N. Dunlap St, Memphis, TN USA; Department of Rheumatology, Le Bonheur Children’s Hospital/University of Tennessee Health Science Center, 50 N. Dunlap St, Memphis, TN USA; Department of Cardiovascular Surgery, Le Bonheur Children’s Hospital/University of Tennessee Health Science Center, 50 N. Dunlap St, Memphis, TN USA

**Keywords:** Hemoptysis, Lobectomy, Extracorporeal membrane oxygenation (ECMO), Plasmapheresis, Diffuse alveolar hemorrhage (DAH), Systemic lupus erythematosus (SLE)

## Abstract

Diffuse alveolar hemorrhage (DAH) from systemic lupus erythematosus (SLE) is a rare but potentially life-threatening condition. We report the case of a 14-year-old female with SLE who developed hypoxia and shock secondary to severe alveolar hemorrhage. She was successfully managed by placement on extracorporeal membrane oxygenation (ECMO) followed by emergent pulmonary lobectomy and medical treatment including high-dose methylprednisolone, cyclophosphamide, intravenous immunoglobulin, and plasmapheresis.

## Background

**Figure 1:**
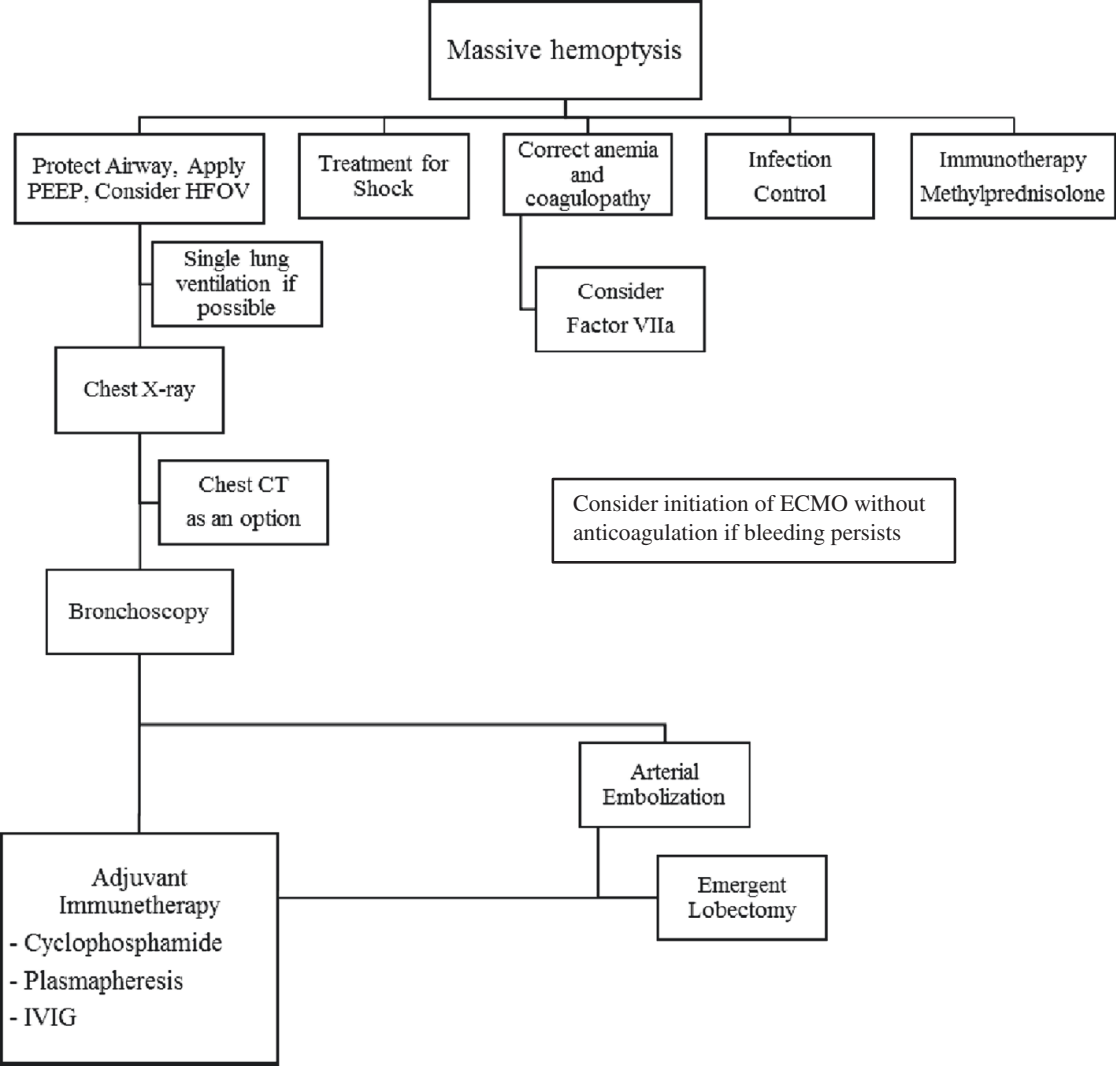
**Management of severe diffuse alveolar hemorrhage with SLE.** Consider initiation of ECMO without anticoagulation if bleeding persists.

A recent report of seven cases of pediatric systemic lupus erythematosus (SLE) with pulmonary hemorrhage had a survival rate of 87.5% [1]; however, the survival rate for diffuse alveolar hemorrhage (DAH) is extremely low when it leads to acute catastrophic hemoptysis with impairment in oxygenation, ventilation, or hemodynamic instability. In adults, high Acute Physiology and Chronic Health Evaluation II scores, requirement of mechanical ventilation, renal failure, and thrombocytopenia are associated with higher mortality in DAH with SLE [2]. Despite recent advances in therapy, mortality in adults has not improved (48% overall survival versus 53% survival in reports published since 1993) using conventional therapies [3]. We experienced a pediatric patient recently diagnosed with SLE presenting in a critically ill condition due to DAH in hypoxic respiratory failure. This patient was managed successfully with combined treatment of interventional catheterization, extracorporeal membrane oxygenation (ECMO) support, pulmonary lobectomy, and early initiation of immunotherapy (Figure 1).

## Case presentation

A 14-year-old African-American female presented with sudden onset of shortness of breath and palpitations. Her past history was significant for a recent diagnosis of SLE when she had presented with complaints of rapid weight loss (9 kg over 2 months), easy fatiguability, and a malar facial rash a month back. Her laboratory evaluation was positive for fluorescent antinuclear antibody (FANA) of 1:360 (speckled pattern), a low white blood cell (WBC) count of 3,300/mm^3^, a low hemoglobin count of 3.3 g/dl, and urine analysis that demonstrated 2+ albumin with 2+ blood. Other pertinent labs included a positive anti-SSA of 311 RU/ml, a positive anti-SSB of 644 RU/ml, and a positive RPR of 1:8 together with a negative test for treponema pallidum. These abnormal labs together with the clinical presentation led to the diagnosis of SLE. She had been started on hydroxychloroquine and prednisone. She was seen in the emergency department (ED) on three different occasions since diagnosis for various non-specific complaints including dry cough, mild hemoptysis, shortness of breath, and syncope. She was found to be severely anemic (hemoglobin of 4.1 g/dl) and treated with blood transfusion and iron supplements. A diagnosis of right lower lobe pneumonia had been made and she was treated with oral antibiotics.

At her recent ED presentation for shortness of breath, chest x-ray showed infiltrates and effusion over the right middle and lower lobes. While in the ED, she developed worsening tachycardia, tachypnea, and hypotension requiring rapid intubation with a single lumen endotracheal tube (ETT). Copious bright red blood was seen upon intubation indicative of massive pulmonary hemorrhage. She was emergently taken to the catheterization laboratory to locate and potentially embolize the source of the bleeding. Selective angiography demonstrated the right bronchial artery as the source of bleeding. However, the anterior spinal artery was found to branch off the right bronchial artery, just proximal to the level of the bleeding. Hence, embolization was deferred due to the potential risk of paraplegia. During the procedure, the patient developed progressive hypoxic respiratory failure requiring escalation of support to high-frequency oscillatory ventilator (HFOV). However, despite a mean air pressure of 45 cm H_2_O and addition of inhaled nitric oxide, there was no improvement. Hence, she was emergently cannulated for venovenous-ECMO. Initially one double lumen cannula was placed in the right internal jugular vein; however, the venous flow was not sufficient. The other cannula was placed in the left femoral vein to achieve the target ECMO flow. The single lumen ETT was exchanged for a double lumen ETT to protect the non-bleeding lung. She was transferred to the operation room on ECMO where she underwent an emergent right upper and middle pulmonary lobectomy. During procedure, the entire right lung was noted to be densely consolidated with a hemorrhagic appearance. Microscopically, the tissue demonstrated diffuse interseptal capillary wall damage with marked neutrophil infiltration and massive intraalveolar hemorrhage, and hemosiderin-laden macrophages are present in all lobes of the right lung. Pathology diagnosis is pulmonary neutrophilic capillaritis with diffuse parenchyma hemorrhage.

**Figure 2:**
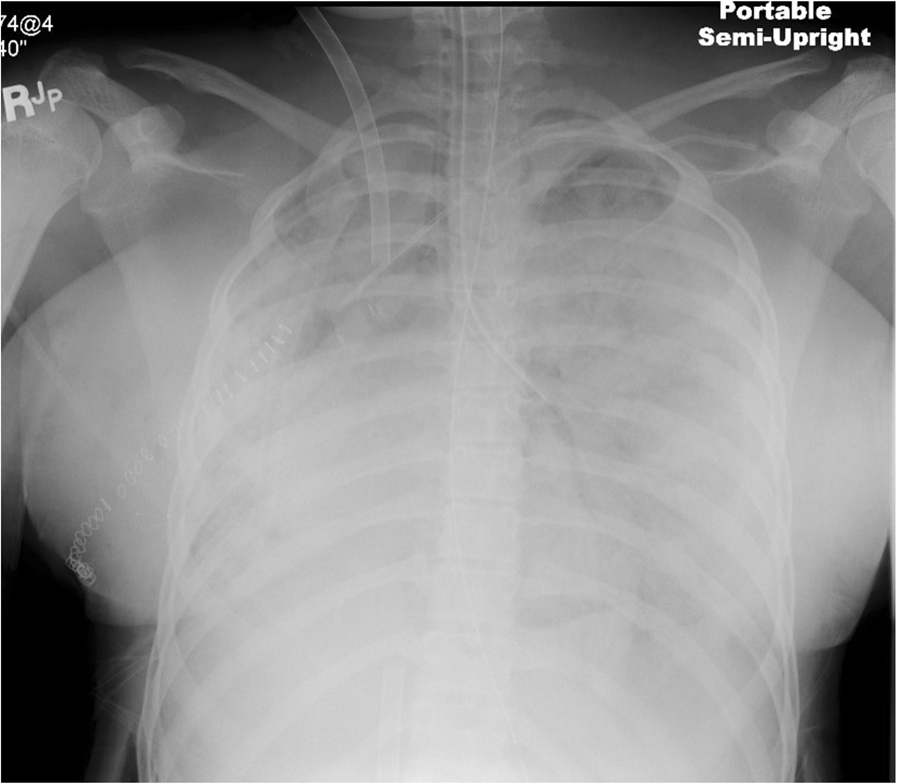
**Patient chest x-ray.** Patient lungs are shown to be clear.

Following surgery, she was transferred to the cardiac ICU where she was maintained on venovenous-ECMO and HFOV due to hypoxia and hemodynamic instability (Figure 2). To avoid further bleeding, platelet was transfused to keep platelet counts >100,000/μl. Anticoagulation therapy with heparin drip for ECMO was not given at the beginning. It was started at the lower dosage to titrate the activated clotting time (ACT) between 140 and 160 s for the first 48 h after the operation, then increased to keep ACT 180–200 s. During the first week, she received high-dose methylprednisolone, 1 g daily for 3 days, which was then reduced to 60 mg every 6 h. In view of ongoing hemoptysis and potential for further pulmonary hemorrhage, she was treated with plasmapheresis while on ECMO with alternate day treatment regimens for 3 cycles. In addition, intravenous immunoglobulin (IVIG) of 2 g/kg/dose was also administered to minimize the risk of recurrent pulmonary hemorrhage. A single dose of 750 mg of cyclophosphamide was also administered to reduce the inflammatory effects of active SLE. Although she received multiple packed red blood cell, frozen fresh plasma, and platelet transfusions during her hospital course, the requirement for blood products declined as medical treatments for SLE with plasmapheresis and IVIG were advanced. A respiratory culture of *streptococcus viridans* was treated with appropriate antibiotics. A chest tube was placed on day 3 for a left hemothorax. On the fifth postoperative day, bronchoscopy demonstrated clear airway without active signs of hemorrhage. The patient was transitioned to a conventional ventilator from the HFOV on post-op day 7. She was successfully decannulated from ECMO on day 10. She was extubated on day 18 and discharged home in a stable condition after 57 days of hospital stay. At the time of discharge, she was comfortably breathing room air without any neurological sequelae. Predischarge chest x-ray revealed clear lung fields.

### Discussion

Although there is a wide spectrum of pulmonary manifestations in SLE, pulmonary hemorrhage occurs in 5 to 10% of pediatric SLE patients with pulmonary involvement. It can present as a chronic low-grade hemorrhage with few early symptoms, but more frequently it presents as acute respiratory distress [4]. One type of pulmonary hemorrhage, DAH, is a rare and life-threatening complication of SLE associated with high mortality [5]. DAH may present in the early course of SLE in children [1] and is associated with high disease activity [6]. Bleeding into the alveolar spaces characterizes the syndrome of DAH and is due to disruption of the alveolar-capillary basement membrane caused by injury or inflammation of the arterioles, venules, or alveolar septal (alveolar wall or interstitial) capillaries.

Thoracic surgery should be consulted early in the course of management, and emergency lung resection is a life-saving procedure if bronchial artery embolization (BAE) is unsuccessful [7]. With advances in interventional radiology, the number of emergent surgery for massive hemoptysis has decreased because of associated morbidity and mortality; however, surgery is indicated for technically difficult cases of BAE, respiratory or hemodynamically unstable patients for BAE, and early or repeated recurrence of hemoptysis after BAE [8,9]. In general, surgery was not considered as an option for patients with moderate to severe lung function impairment. However, early initiation of ECMO could improve oxygen delivery and allow to perform a life-saving surgery as seen in our case [8].

ECMO was used as a rescue therapy for the severe respiratory failure in DAH secondary to SLE [10]. Anticoagulation therapy with heparin was initially deferred until hemostasis was achieved, despite a risk of ECMO circuit thrombosis, since ECMO can be performed without systemic anticoagulation for short periods [11,12]. Once hemostasis is achieved, anticoagulation therapy should be initiated to prevent the thrombosis in the ECMO circuit and oxygenation membrane. Kolovos et al. reported that eight children with respiratory failure from pulmonary hemorrhage were successfully managed with ECMO with continuous heparin drip with the target of ACT 160–180 s [13]. Currently the ideal target level of anticoagulation for patients with pulmonary hemorrhage in children is unknown.

These interventions would not have been successful if medical treatment against SLE was not initiated as soon as possible. The main therapy for DAH with SLE in children and adults is high-dose methylprednisolone [1] combined with cyclophosphamide [6] and plasmapheresis [5]. The other therapeutic options are rituximab, IVIG, and mycophenolate mofetil [6]. Plasmapheresis performed on ECMO has a benefit for sharing vascular access and has been used for severe pulmonary hemorrhage due to other autoimmune diseases [14]. Currently there are no therapeutic guidelines for this life-threatening manifestation of SLE due to the rarity of this condition. Multi-institutional data collection would be beneficial for determining the effectiveness of each therapeutic options for this condition.

## Conclusion

This is the first report of a child with massive hemoptysis due to SLE requiring ECMO support followed by emergency lobectomy and early initiation of immunotherapy including plasmapheresis that resulted in a good outcome. Although ongoing hemorrhage might be considered a contraindication for ECMO, our case demonstrated that this is an effective intervention. The successful outcome of our patient suggests that cases of SLE with DAH unresponsive to conventional therapies can be successfully treated with combinations of interventional radiology, ECMO, surgery, and immunotherapy.

## Consent

Informed consent was obtained from the family for the publication of this case report and any accompanying images. A copy of the written consent is available for review by the Editor-in-Chief of this journal. We obtained the institutional review board approval (the University of Tennessee Health Science Center) for this study including a waiver of the need for informed consent from the patient.

## Abbreviations

DAH: Diffuse alveolar hemorrhage
SLE: Systemic lupus erythematosus
ECMO: Extracorporeal membrane oxygenation
FANA: Fluorescent antinuclear antibody
WBC: White blood cell
ED: Emergency department
ETT: Endotracheal tube
HFOV: High-frequency oscillatory ventilation
ICU: Intensive care unit
IVIG: Intravenous immunoglobulin
BAE: Bronchial artery embolization
